# Docosahexaenoic acid induces the degradation of HPV E6/E7 oncoproteins by activating the ubiquitin–proteasome system

**DOI:** 10.1038/cddis.2014.477

**Published:** 2014-11-13

**Authors:** K Jing, S Shin, S Jeong, S Kim, K-S Song, J-H Park, J-Y Heo, K-S Seo, S-K Park, G-R Kweon, T Wu, J-I Park, K Lim

**Affiliations:** 1 Department of Biochemistry, School of Medicine, Chungnam National University, Daejeon, Korea; 2 Infection Signaling Network Research Center, Chungnam National University, Daejeon, Korea; 3 Stem Cell Research and Cellular Therapy Center, Affiliated Hospital of Guangdong Medical College, Zhanjiang, China; 4 Department of Pathology and Laboratory Medicine, Tulane University School of Medicine, New Orleans, LA, USA; 5 Cancer Research Institute, Chungnam National University, Daejeon, Korea

## Abstract

The oncogenic human papillomavirus (HPV) E6/E7 proteins are essential for the onset and maintenance of HPV-associated malignancies. Here, we report that activation of the cellular ubiquitin–proteasome system (UPS) by the omega-3 fatty acid, docosahexaenoic acid (DHA), leads to proteasome-mediated degradation of E6/E7 viral proteins and the induction of apoptosis in HPV-infected cancer cells. The increases in UPS activity and degradation of E6/E7 oncoproteins were associated with DHA-induced overproduction of mitochondrial reactive oxygen species (ROS). Exogenous oxidative stress and pharmacological induction of mitochondrial ROS showed effects similar to those of DHA, and inhibition of ROS production abolished UPS activation, E6/E7 viral protein destabilization, and apoptosis. These findings identify a novel role for DHA in the regulation of UPS and viral proteins, and provide evidence for the use of DHA as a mechanistically unique anticancer agent for the chemoprevention and treatment of HPV-associated tumors.

## Introduction

Oncogenic human papillomavirus (HPV) is tightly linked to the development of nongenital and genital malignancies. Of the oncogenic HPVs, HPV-16 and HPV-18 are the most commonly detected genotypes in malignant biopsies.^1^ During the progression of HPV-associated cancers, the entire or fragments of HPV genome randomly integrates into the host cell chromatin DNA, leading to the constitutive expression of two early viral proteins: E6 and E7.^2^ As the main transforming proteins produced by oncogenic HPVs, E6/E7 primarily contributes to carcinogenesis through their modulation of pivotal cell signaling components, including the tumor suppressors p53 and retinoblastoma (Rb).^3, 4^ Whereas E6 targets p53 for degradation, impairing its growth-inhibitory and apoptosis-inducing effects, E7 inactivates Rb, thereby promoting cell cycle progression.^5^ As sustained inactivation of p53 and Rb favors deregulated cell growth, E6/E7 are attractive therapeutic candidates for the treatment of HPV-associated tumors.^6^ Indeed, inhibition of E6/E7 restores the function of the p53 and Rb, thereby eliciting growth arrest and death in cells infected with HPV-16/18.^7, 8, 9^

HPV E6/E7 are rapidly degraded by the ubiquitin–proteasome system (UPS).^10, 11, 12, 13^ In the UPS, substrate proteins are first ubiquitinated through a sequential enzymatic cascade, and the ubiquitinated conjugates (Ub-conjugates) are then degraded by the proteasome.^14^ These events are equally significant, and blockade of any of them can impair the degradation of UPS substrate proteins.^15^ Moreover, in addition to degrading ubiquitinated proteins, the proteasome is responsible for the proteolysis of certain nonubiquitinated proteins, such as ornithine decarboxylase (ODC).^14, 16^ Key UPS components, including the proteasome and all classes of enzymes involved in ubiquitination, are redox-sensitive,^17^ and ubiquitination and proteasomal degradation of UPS substrates have been linked to reactive oxygen species (ROS). For instance, in response to a variety of ROS inducers, the enzymatic activities of the ubiquitin-conjugation system are enhanced, resulting in increased level of Ub-conjugates,^18, 19^ and ROS inhibition impairs proteasome activity in cancer cells;^20^ these findings suggest that ROS may have a positive impact on cellular UPS function.

Docosahexaenoic acid (DHA), the most unsaturated omega-3 fatty acid, has been demonstrated to possess pro-apoptotic activity against tumor cells.^21, 22^ We have also reported that DHA triggers apoptosis in SiHa cancer cells expressing HPV-16, which could be partially rescued by proteasome inactivation,^23^ suggesting that the UPS is involved in the DHA-triggered death of oncogenic HPV-infected cells. Here, we characterized the molecular mechanism underlying the UPS-associated anticancer activity of DHA in HPV-positive cancer cells using GFP-based UPS reporter substrates. The intracellular steady-state levels of these reporters, including GFP-CL1 (a 16-amino-acid CL1 degradation signal peptide fused to the carboxy terminus of GFP)^16^ and Ub^G76V^-GFP (a mutated noncleavable ubiquitin moiety fused to the amino terminus of GFP)^24^ reflect the overall flux through the UPS; interfering with the function of the UPS leads to their accumulation.^15^ Our results show that DHA promoted E6/E7 degradation through ROS-mediated UPS activation, thereby inducing apoptosis in HPV-infected cancer cells. This is the first study to provide evidence for the DHA-induced post-translational regulation of viral oncoproteins, and the findings lead to a better understanding of how DHA affects UPS.

## Results

### DHA-induced apoptosis in HPV-infected cancer cells involves E6/E7 repression

**Figure 1:**
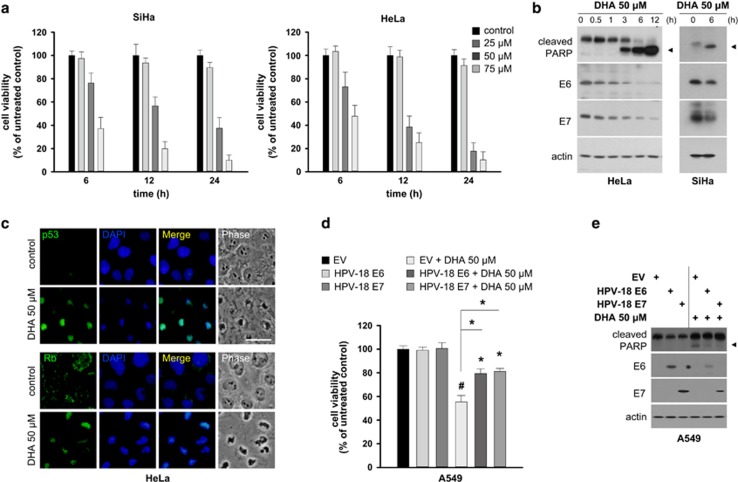
DHA-induced E6/E7 repression is associated with apoptosis in oncogenic HPV-infected cancer cells. (**a**) SiHa (left) and HeLa (right) cells were incubated with increasing concentrations (0, 25, 50 and 75 *μ*M) of DHA for 6, 12 and 24 h, and cell viability was measured by MTT assays. (**b**) HeLa (left) and SiHa (right) cells were incubated with 50 *μ*M DHA for the indicated times, and the expression levels of PARP and E6/E7 were assessed by western blotting. (**c**) Phase-contrast and merged images of HeLa cells treated with vehicle or 50 *μ*M DHA for 6 h and stained for p53 (top) and Rb (bottom) (green). Nuclei were stained with DAPI (scale bar, 10 *μ*m). (**d** and **e**) A549 cells seeded on 96-well plates or 10-cm dishes were transiently transfected with a control empty vector (EV) or with HPV-18 E6/E7 expression vectors. At 36 h post transfection, the cells were treated, or not, with 50 *μ*M DHA for 6 h and then subjected to MTT assays (**d**) or western blotting (**e**). Results are represented as mean±S.D. values. Error bars indicate S.D. (*n*=3). *P<0.05; ^#^*P*<0.001

DHA induces apoptosis in SiHa cells infected with HPV-16.^23^ To determine whether DHA affects the fate of cells infected with other oncogenic HPV types, we compared the viability and morphological alterations in HeLa cells expressing HPV-18 and SiHa cells treated with DHA. DHA reduced the viability of both HeLa and SiHa cells (Figure 1a), and induced similar morphological changes in the two cell lines (Supplementary Figure 1a). In addition, there was a marked increase in the number of HeLa cells harboring nuclear fragments and DNA strand breaks (Supplementary Figures 1b and c) after exposure to DHA. The expression of two common apoptotic molecular markers, cleaved caspase 3 and poly(ADP-ribose) polymerase (PARP), was upregulated in HeLa cells incubated with DHA, but not in cells incubated with other highly unsaturated fatty acids including eicosapentaenoic acid and arachidonic acid (Supplementary Figure 1d). These findings establish that apoptosis is the mode of death caused by the specific type of omega-3 fatty acid, DHA, in cancer cells expressing oncogenic HPV.

E6/E7 have an important role in maintaining the malignant phenotype of HPV-infected cancer cells;^3^ therefore, we next examined the effect of DHA on E6/E7. Although DHA led to increases in cleaved PARP in HeLa and SiHa cells, it reduced the levels of E6/E7 (Figure 1b). Inhibiting E6/E7 restores the functions of their primary cellular targets, p53 and Rb.^7^ Consistent with this, whereas the nuclear staining for p53/Rb was almost undetectable in control cells, it was markedly increased in DHA-treated HeLa cells (Figure 1c). Continuous E6/E7 expression is essential for the survival of HPV-infected cancer cells.^5^ As our results indicate the involvement of E6/E7 repression in the DHA-induced apoptotic process, we hypothesized that DHA might induce apoptosis by suppressing E6/E7. If this were the case, the introduction of E6 or E7 into cells should attenuate DHA-induced apoptosis. Indeed, the effects of DHA on cytotoxicity and the expression of cleaved PARP (Figures 1d and e) were markedly reduced when HPV-18 E6 or E7 was transiently expressed in HPV-deficient A549 cancer cells. These data indicate that DHA attenuates E6/E7, thereby promoting apoptosis in HPV-infected cancer cells.

### DHA stimulates the UPS-dependent E6/E7 degradation

**Figure 2:**
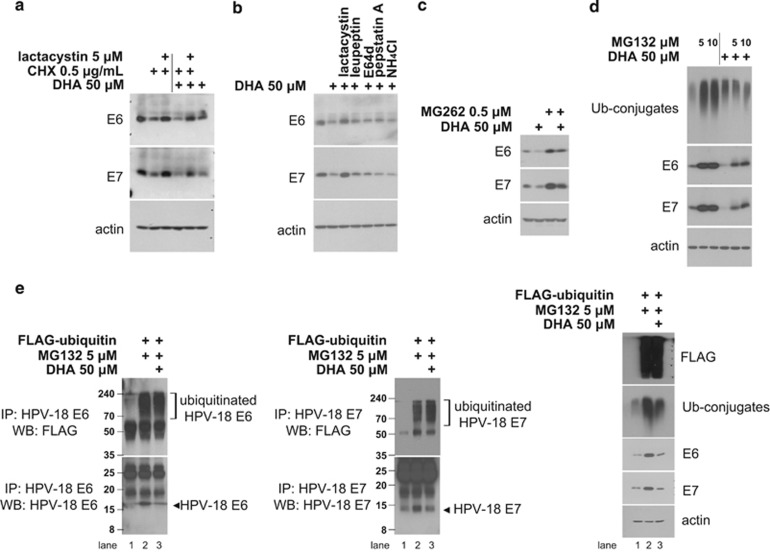
DHA induces the UPS-dependent degradation of E6/E7 viral oncoproteins. (**a**) HeLa cultures were pretreated, or not, with lactacystin (5 *μ*M) for 1 h before the addition of CHX (0.5 *μ*g/ml), DHA (50 *μ*M) or a combination of the two. Cells were collected 6 h later and subjected to western blot analysis. (**b**) HeLa cells were left untreated or treated with 50 *μ*M DHA for 6 h with 1 h of pretreatment of 5 *μ*M lactacystin, 10 *μ*M leupeptin, 2.5 *μ*g/ml E64d, 1 *μ*M pepstatin A and 10 mM NH_4_Cl, respectively. Whole-cell lysates were extracted and blotted with antibodies against E6/E7. (**c**) HeLa cells were preincubated with or without 0.5 *μ*M MG262 for 1 h before the addition of 50 *μ*M DHA. After 6 h, whole-cell lysates were blotted with anti-E6/E7 antibodies. (**d**) HeLa cells were pretreated, or not, with 5 *μ*M or 10 *μ*M MG132 for 1 h and then treated with 50 *μ*M DHA for 6 h. The expression levels of indicated proteins were then examined by western blotting (WB). (**e**) DHA increases E6/E7 ubiquitination. HeLa cells transiently expressing a control vector or FLAG-ubiquitin plasmid were pretreated, or not, with 5 *μ*M MG132 for 1 h and then treated with 50 *μ*M DHA for 6 h. Whole-cell lysates were subjected to immunoprecipitation (IP) with anti-E6 (left) or -E7 (middle) antibodies, as indicated, followed by WB with anti-FLAG (top) or anti-E6/E7 (bottom) antibodies. Right, whole-cell lysates were blotted with anti-FLAG, -ubiquitin (Ub-conjugates) and -E6/E7 antibodies to examine expression levels

Because E6/E7 undergo proteasomal degradation,^12, 13^ we inactivated the proteosome using lactacystin and examined the effects on the DHA-mediated regulation of E6/E7 in HeLa cells preincubated with or without cycloheximide (CHX), a translation inhibitor. Lactacystin abolished the CHX-induced E6/E7 depletion and prevented the inhibitory effects of CHX plus DHA on E6/E7 (Figure 2a). The ability of lactacystin to prevent the reduction in E6/E7 appeared to be a specific consequence of proteasome inhibition, as the lysosome inhibitor NH_4_Cl and various protease inhibitors, including leupeptin, E64d and pepstatin A, had only a slight (or no) impact (Figure 2b). These observations suggest that DHA downregulates E6/E7 at the post-translational level by accelerating their proteasomal degradation. To confirm this, HeLa cells were exposed to another two different classes of proteasome inhibitors, MG262 or MG132,^15^ before DHA treatment and E6/E7 levels were monitored (Figures 2c and d). Treatment with MG262 and MG132 led to a marked increase in the baseline levels of E6/E7, confirming that E6/E7 is degraded by the proteasome. Furthermore, similar to lactacystin, these inhibitors also blocked the loss of E6/E7 caused by DHA; however, the block was not complete, as cells cotreated with DHA and MG262 (or MG132) showed lower levels of E6/E7 than those treated with inhibitors alone.

Proteins are generally ubiquitinated prior to degradation by the proteasome.^14, 15^ Therefore, we examined the effect of DHA on the ubiquitination status of E6/E7, assuming that increased ubiquitination in the presence of a proteasome inhibitor would indicate that DHA-induced degradation is mediated by ubiquitination. HeLa cells were transiently transfected with a control vector or a plasmid encoding FLAG-tagged ubiquitin, and then treated with DHA in the presence of MG132. E6/E7 ubiquitination was then assessed by immunoprecipitation of E6/E7 followed by western blotting with anti-FLAG antibodies (Figure 2e). Consistent with our previous observations, MG132 did not completely prevent DHA-induced decreases in E6/E7 (compare lane 2 with lane 3). However, despite the lower levels of E6/E7 in cells exposed to DHA plus MG132, the amounts of ubiquitinated E6/E7 in these cells were comparable with those in cells treated with MG132 alone. Therefore, the relatively higher levels of ubiquitinated E6/E7 in cells cotreated with DHA and MG132 suggest that DHA triggers E6/E7 ubiquitination.

### DHA-induced E6/E7 degradation is associated with enhanced UPS function

**Figure 3:**
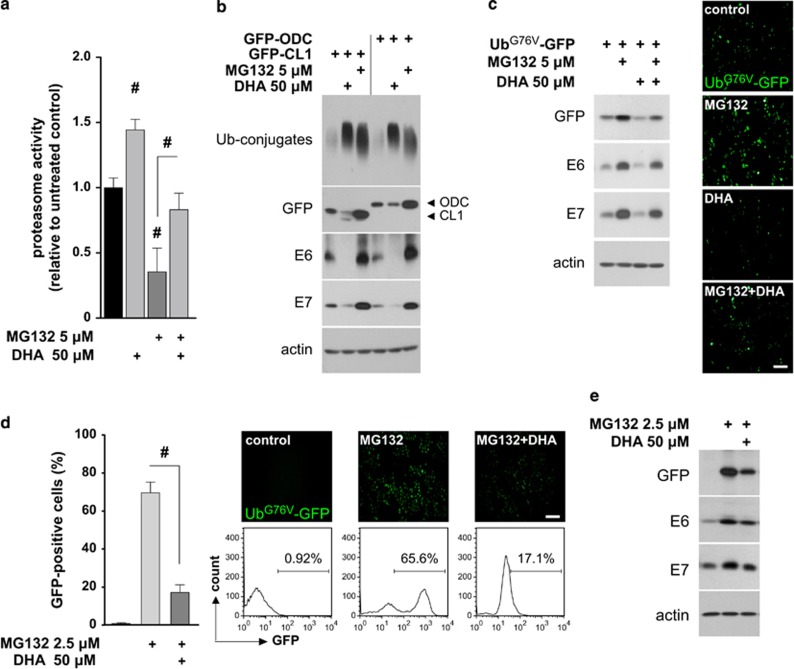
DHA promotes the degradation of E6/E7 viral proteins by increasing UPS activity. (**a**) HeLa cells were left untreated or pretreated with 5 *μ*M MG132 for 1 h, and then exposed to 50 *μ*M DHA for 6 h. Total cell extracts (10 *μ*g) were prepared and incubated with fluorogenic peptide Suc-LLVY-aminomethylcoumarin for 1 h, and the fluorescence signal was measured. The untreated control was set to 1. (**b**) HeLa cells transiently transfected with the indicated reporters were incubated with 50 *μ*M DHA or 5 *μ*M MG132 for 6 h, and the expression of the reporters, E6/E7, and ubiquitinated conjugates (Ub-conjugates) was examined by western blotting. (**c**) HeLa cells transiently transfected with the Ub^G76V^-GFP reporter were pretreated, or not, with 5 *μ*M MG132 for 1 h and then treated with 50 *μ*M DHA for 6 h. Left, expression levels of the reporters and E6/E7 were analyzed by western blotting; right, fluorescence micrographs of Ub^G76V^-GFP reporter expression following different treatments (as described in the left panel). Scale bar, 100 *μ*m. (**d** and **e**) DHA increases the degradation of the Ub^G76V^-GFP reporter and the E6/E7 proteins. Ub^G76V^-GFP HeLa cells were either left untreated or preincubated with 2.5 *μ*M MG132 for 4 h to induce the accumulation of the reporter. The cells were then treated, or not, with 50 *μ*M DHA for 6 h. Cells were collected and GFP fluorescence was examined by flow cytometry (**d**). The bar graph shows the results of four independent experiments. Fluorescence images and histograms show a representative experiment (scale bar, 100 *μ*m). Alternatively, the cells were subjected to immunoblotting (**e**). Data are represented as mean±S.D. values, and error bars indicate S.D. (*n*⩾3). ^#^*P*<0.001

Although DHA and proteasome inhibitor MG132 both increased Ub-conjugates levels (Figure 2d), they had opposite effects on proteasome activity (Figure 3a), and the Ub-conjugates accumulation and proteasome inhibition induced by MG132 could be partially reversed by DHA. This suggests that the formation of Ub-conjugates caused by MG132 and DHA may be differentially mediated by their effects on UPS. To test this, and to ascertain whether DHA-induced E6/E7 degradation also relates to the UPS, HeLa cells were transiently transfected with a UPS reporter, GFP-CL1, or a proteasome activity reporter, GFP-ODC,^16^ and the effects of MG132 and DHA on the expression of these reporters, E6/E7, and Ub-conjugates were compared (Figure 3b). Whereas MG132 increased the expression of both GFP-ODC and GFP-CL1, their expression was reduced by DHA. In addition, the well-documented proteasomal substrate, p53 was suppressed in response to long-term DHA treatment, which could be rescued by MG132 (Supplementary Figure 2). These findings indicate that, unlike MG132, DHA enhances proteasome activity and increases the expression of Ub-conjugates without blocking UPS, and that DHA-induced E6/E7 degradation is concomitant with increased UPS function.

To confirm these findings and to explore the relationship between the observed increases in E6/E7 degradation and UPS activity in response to DHA, HeLa cells transiently expressing another UPS reporter, Ub^G76V^-GFP,^24^ were pretreated with or without MG132 prior to DHA exposure (Figure 3c). DHA decreased the basal levels of Ub^G76V^-GFP and E6/E7. Meanwhile, the levels of the Ub^G76V^-GFP reporter and E6/E7 in cells exposed to MG132 alone were higher than those in cells treated with MG132 plus DHA. These findings confirmed the result obtained with the GFP-CL1 reporter, arguing that DHA simultaneously induces UPS activation and the UPS-dependent E6/E7 degradation. Notably, because HeLa cells were preincubated with MG132, UPS function within the cells was partially inhibited prior to DHA treatment. Therefore, the observation that cells cotreated with MG132 and DHA showed a lower level of Ub^G76V^-GFP reporter expression relative to that in MG132-treated cells suggests that DHA (to a certain extent) overrides the inhibitory effect of MG132 on UPS. This was further confirmed in HeLa cells stably expressing the Ub^G76V^-GFP reporter (Ub^G76V^-GFP HeLa). The Ub^G76V^-GFP reporter does not accumulate in stable transfectants in the absence of UPS inhibition because of its highly efficient degradation by the UPS.^24^ As shown in Figure 3d, the MG132-induced accumulation of the Ub^G76V^-GFP reporter was significantly inhibited when DHA was added to the medium. Likewise, the MG132-induced increases in E6/E7 were also inhibited by DHA (Figure 3e). These data demonstrate that DHA-induced cellular UPS activation is, at least partially, responsible for the degradation of E6/E7.

### ROS are associated with the reduction in DHA-induced E6/E7

**Figure 4:**
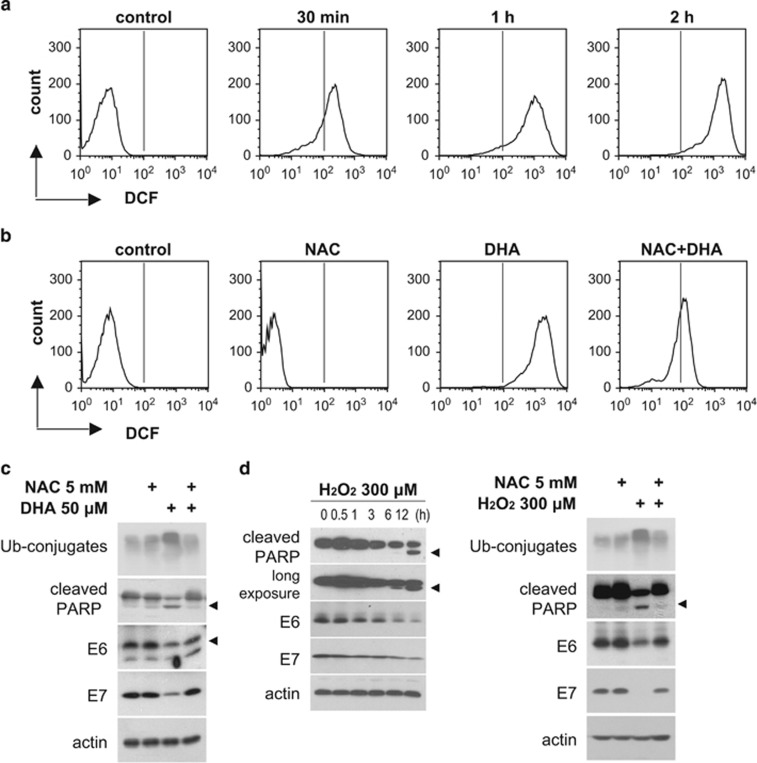
The DHA-induced reduction in E6/E7 expression is dependent on ROS accumulation. (**a**) HeLa cells were treated with 50 *μ*M DHA for the indicated times, and intracellular ROS levels were detected by flow cytometry using CM-H2DCFDA probes. (**b**) CM-H2DCFDA-loaded HeLa cells were pretreated, or not, with 5 mM NAC for 1 h followed by 50 *μ*M DHA for 2 h, and ROS levels were examined by flow cytometry. (**c**) HeLa cells were pretreated, or not, for 1 h with 5 mM NAC followed by 50 *μ*M DHA for 6 h. Whole-cell lysates were blotted with the indicated antibodies. (**d**) HeLa cells were incubated with 300 *μ*M H_2_O_2_ for the indicated times (left), or pretreated, or not, for 1 h with 5 mM NAC followed by incubation with 300 *μ*M H_2_O_2_ for 6 h (right). Whole-cell lysates were extracted, and blotted with the indicated antibodies

DHA triggers ROS accumulation in tumor cells,^22^ and has been shown to selectively inhibit the growth of oncogenic HPV-immortalized keratinocytes via lipid peroxidation.^25^ Therefore, we examined the potential involvement of ROS in the DHA-induced E6/E7 downregulation. Flow cytometric analysis using a general ROS-sensitive probe, CM-H2DCFDA, for total cellular ROS measurement showed a time-dependent increase in dichlorodihydrofluorescein (DCF) fluorescence in HeLa and SiHa cells exposed to DHA (Figure 4a; Supplementary Figure 3a). The ROS scavenger, *N*-acetyl-cysteine (NAC), markedly attenuated the DHA-induced DCF fluorescence (Figure 4b; Supplementary Figure 3b) and prevented the reduction in E6/E7 and cleaved PARP levels induced by DHA and by exogenous hydrogen peroxide (H_2_O_2_) (Figures 4c and d; Supplementary Figure 3c). Furthermore, preincubation of HeLa cells with other antioxidants (EUK8 and sodium pyruvate) also inhibited DHA-mediated effects on cell death and E6/E7 expression (Supplementary Figure 3d). These observations suggest that DHA induces the production of ROS, which regulate E6/E7 and apoptosis.

### Decreased E6/E7 expression levels caused by DHA involve mitochondrial ROS overproduction

**Figure 5:**
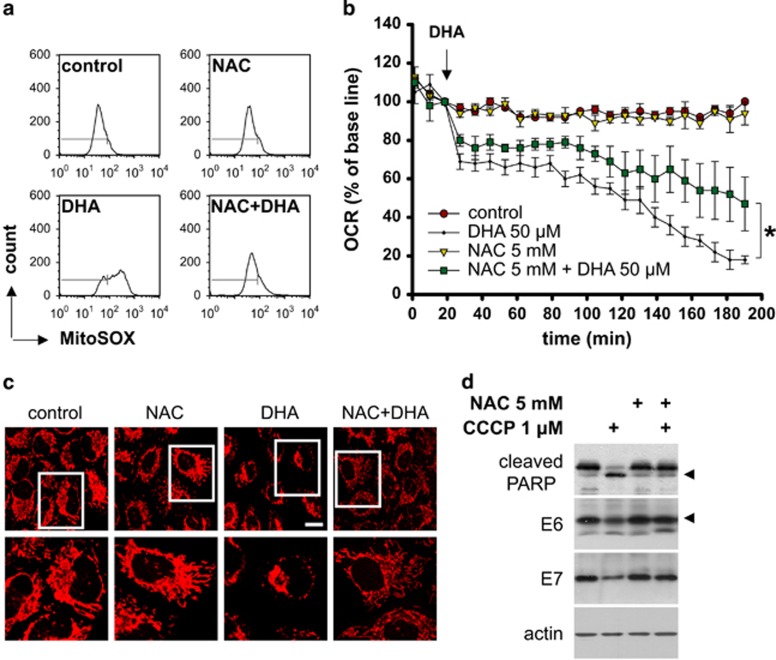
Involvement of mitochondrial ROS in the downregulation of E6/E7. (**a**) HeLa cells were pretreated, or not, for 1 h with 5 mM NAC followed by 50 *μ*M DHA for 2 h, and mitochondrial ROS levels were examined by flow cytometry using MitoSOX Red probes (Molecular Probes). (**b**) The oxygen consumption rate (OCR) of HeLa cells treated with 5 mM NAC, 50 *μ*M DHA, or NAC plus DHA was measured using a Seahorse Bioscience XF Analyzer. The arrow indicates the time of DHA addition. (**c**) Representative confocal microscopic images of HeLa cells stained with a vital mitochondrial dye (MitoTracker Red) after incubation with NAC, DHA, or NAC plus DHA. Cells were preincubated with 5 mM NAC for 1 h, and then treated with or without 50 *μ*M DHA for another 2 h. The bottom panel indicates higher power views of the boxed area in the top panel (scale bar, 10 *μ*m). (**d**) HeLa cells were preincubated with 5 mM NAC for 1 h, and then treated with or without 1 *μ*M CCCP for another 6 h. The protein levels of E6/E7 and PARP were analyzed by western blotting. Mean±S.D. values are shown, and error bars indicate S.D. (*n*=3). *P<0.05

Preliminary experiments identified marked DCF fluorescence in HeLa cells as early as 20 min after DHA treatment; this fluorescence primarily colocalized with a vital mitochondrial dye, MitoTracker Red (Molecular Probes, Eugene, OR, USA) (Supplementary Figures 4a and b), implying that mitochondria are a likely source of the ROS induced by DHA. Detection of mitochondrial ROS using MitoSOX Red revealed that DHA caused the increase in mitochondria-derived ROS production, which was inhibited by NAC (Figure 5a). Excessive mitochondrial ROS production is often accompanied by loss of mitochondrial membrane potential and mitochondrial membrane non-oxidized cardiolipin, both of which are important for the maintenance of mitochondrial function.^26^ In line with this, DHA reduced the levels of mitochondrial membrane potential and non-oxidized cardiolipin, and these effects of DHA were markedly reversed by NAC (Supplementary Figure 4c). To provide further evidence for the involvement of mitochondria in DHA-induced ROS production, we compared mitochondrial function in HeLa cells treated with DHA and/or NAC by monitoring changes in oxygen consumption rates (OCRs). The result showed that NAC inhibited the DHA-mediated time-dependent decrease in the OCR (Figure 5b). Confocal microscopy analysis of cells stained with MitoTracker Red 2 h after treatment confirmed that NAC prevented the loss of respiring mitochondria caused by DHA (Figure 5c). These data clearly indicate that mitochondrial ROS account for the excessive ROS accumulation in HPV-infected cancer cells exposed to DHA. Having found this, we next sought to evaluate the impact of mitochondrial ROS on E6/E7 more directly. HeLa cells were treated with NAC and/or carbonyl cyanide m-chlorophenylhydrazone (CCCP), a mitochondrial dysfunction inducer that has been shown to trigger ROS overproduction in mitochondria.^27, 28^ CCCP increased mitochondrial ROS production in HeLa cells (Supplementary Figure 4d). Notably, it also reduced E6/E7, and this effect could be blocked by NAC (Figure 5d). These results suggest that mitochondrial ROS affect E6/E7 expression and favor the loss of E6/E7 caused by DHA.

### DHA-induced ROS promote E6/E7 degradation by increasing UPS activity

**Figure 6:**
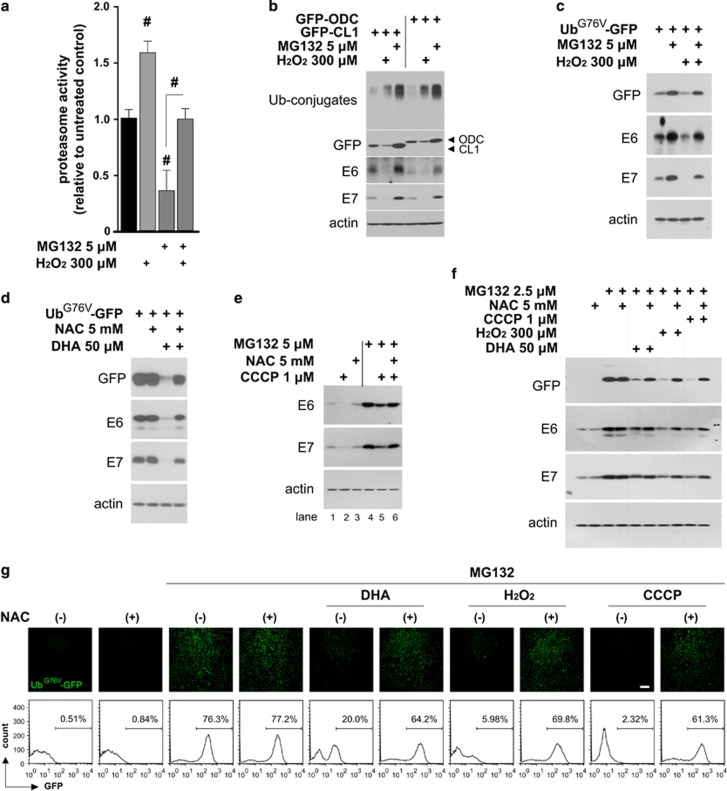
ROS are responsible for the increase in UPS activity and the subsequent degradation of E6/E7. (**a**) HeLa cells were left untreated or pretreated with 5 *μ*M MG132 for 1 h, and then exposed to 300 *μ*M H_2_O_2_ for 6 h. The relative proteasome activity in cell extracts containing 10 *μ*g of proteins was measured. The untreated control was set to 1. (**b**) HeLa cells transiently transfected with the indicated reporters were incubated with 300 *μ*M H_2_O_2_ or 5 *μ*M MG132 for 6 h, and the levels of the reporters, E6/E7, and Ub-conjugates were examined by western blotting. (**c**) HeLa cells transiently expressing the Ub^G76V^-GFP reporter were pretreated, or not, for 1 h with 5 *μ*M MG132 and then incubated with 300 *μ*M H_2_O_2_ for 6 h. GFP and E6/E7 expression levels were then examined by western blotting. (**d**) HeLa cells transiently expressing the Ub^G76V^-GFP reporter were pretreated, or not, for 1 h with 5 mM NAC and then incubated with 50 *μ*M DHA for 6 h. GFP and E6/E7 expression levels were then examined by western blotting. (**e**) HeLa cells were preincubated with or without 5 *μ*M MG132 for 1 h and then exposed to NAC (5 mM), CCCP (1 *μ*M), or a combination of both, for 6 h. Where indicated, NAC was added 1 h before CCCP treatment, and the expression levels of E6/E7 were analyzed by western blotting. (**f** and **g**) Ub^G76V^-GFP HeLa cells were left untreated or preincubated with 2.5 *μ*M MG132 for 4 h, and then treated with NAC (5 mM), DHA (50 *μ*M), H_2_O_2_ (300 *μ*M), CCCP (1 *μ*M), or a combination of NAC and DHA/H_2_O_2_/CCCP for 6 h. Where indicated, NAC was added to the medium 1 h before treatment with DHA, H_2_O_2_, or CCCP. (**f**) Cells were collected and subjected to immunoblotting with the indicated antibodies. (**g**) GFP expression was photographed under a fluorescent microscope (top) and then examined by flow cytometry (bottom). Scale bar, 200 *μ*m. Data are represented as mean±S.D. values, and error bars indicate S.D. (*n*⩾3). ^#^*P*<0.001

ROS are implicated in the regulation of UPS components,^17, 18, 19^ it is thus possible that they may have a causative role in the DHA-induced UPS activation and subsequent E6/E7 degradation. To test this, the effects of exogenous ROS on UPS function and the UPS-mediated E6/E7 downregulation were first assessed. Exposure of HeLa and SiHa cells to H_2_O_2_ elevated the expression level of Ub-conjugates (Figure 4d, right panel; Supplementary Figure 3c, right panel). This accumulation of Ub-conjugates appeared to result from an enhanced cellular ubiquitination event, instead of the proteasome inactivation, because H_2_O_2_-treated cells exhibited significantly improved proteasome activities (Figure 6a), even under conditions of preincubation with MG132. In addition, when HeLa cells transiently transfected with GFP-CL1 or GFP-ODC reporters were incubated with H_2_O_2_ (Figure 6b), the expression levels of these reporters and E6/E7 were diminished. Experiments performed in HeLa cells transiently transfected with the Ub^G76V^-GFP reporter (Figure 6c) further showed that not only H_2_O_2_ did reduce the basal level of Ub^G76V^-GFP and E6/E7, it abolished the MG132-induced increases in Ub^G76V^-GFP reporter and E6/E7. These findings indicate that exogenous ROS accelerate UPS-dependent E6/E7 degradation by increasing UPS activity in our cellular context, and raise the possibility that DHA-induced ROS may have a similar role. Indeed, we found that in HeLa cells transiently expressing the Ub^G76V^-GFP reporter (Figure 6d), NAC-mediated ROS inhibition led to a marked reversal of the DHA-induced reduction in Ub^G76V^-GFP and E6/E7. Moreover, pharmacological mitochondrial ROS inducer, CCCP inhibited the MG132-induced increase in E6/E7 expression (Figure 6e, compare lane 5 with lane 4), whereas NAC blocked this inhibitory effect (compare lane 6 with lane 5), supporting the view that mitochondria-derived ROS also activate UPS and induce UPS-dependent E6/E7 degradation. Further experiments using HeLa cells harboring Ub^G76V^-GFP (Figures 6 f and g) confirmed these results. The MG132-induced accumulation of Ub^G76V^-GFP reporter and E6/E7 was remarkably inhibited by DHA, H_2_O_2_, and CCCP. Importantly, these effects of DHA, H_2_O_2_, and CCCP were partially reversed in the presence of ROS inhibitor NAC. These results indicate that ROS act upstream of the UPS and promote the DHA-induced E6/E7 degradation by increasing UPS function.

## Discussion

The E6/E7 maintain the malignant phenotype of HPV-infected cancer cells and interfere with molecules that take part in the apoptotic pathway.^29^ We showed that DHA induced apoptosis in HeLa and SiHa cells harboring the two most prevalent oncogenic HPV types, and downregulated E6/E7 levels. This finding, together with the observed resistance to DHA-induced apoptosis in HPV-negative cancer cells expressing E6/E7, is consistent with the inhibitory effects of E6/E7 on apoptosis. Furthermore, nuclear p53/Rb is almost undetectable in HPV-infected tumor cells due to their continuous inactivation caused by E6/E7 presence;^30^ this loss of p53/Rb contributes to the anti-apoptotic properties of E6/E7.^9, 31^ In agreement with these previous studies, we found that the DHA-mediated downregulation of E6/E7 and apoptosis induction was associated with increased nuclear staining for p53/Rb. P53/Rb restoration has been characterized as a principal mechanism underlying the E6/E7 repression-mediated apoptosis.^8, 9, 32^ It is thus reasonable to assume that p53/Rb reactivation (triggered by E6/E7 inhibition) is responsible for the DHA-induced apoptotic cell death. However, apart from p53/Rb, molecules including Notch and Sirtuin-1 have also been shown to participate in the apoptosis caused by E6/E7 loss.^33, 34^ An important challenge for future studies thus will be to distinguish between these possibilities and to determine whether the DHA-induced apoptotic process involves a novel target of E6/E7.

DHA promoted the UPS-dependent E6/E7 degradation along with an increase in UPS activity, and perturbation of UPS function inhibited the DHA-induced downregulation of E6/E7, suggesting that the inhibitory effect of DHA on E6/E7 involves UPS activation. This also provides a possible explanation for our previous results,^23^ which show that DHA only transiently upregulates p53 expression in SiHa cells, and that long-term incubation (24 h) of cells with DHA reduces p53 to a level lower than that in untreated cells. This phenomenon of p53 upregulation followed by a prolonged decrease was also observed in HeLa cells (Supplementary Figure 2a). In general, p53 and mouse double minute 2 (MDM2) ubiquitin ligase are mutually regulated by an autoregulatory feedback loop. Activated p53 induces the transcription of MDM2, which in turn targets p53 for proteolytic degradation; however, the MDM2-mediated p53 degradation pathway is switched off in HPV-infected cancer cells because of the persistent degradation of p53 by E6.^31^ As DHA-induced reduction in E6 derives from UPS activation, the observed p53 expression pattern could be attributed to the reactivation of p53, which is mediated by E6 inhibition, and the subsequent further degradation of p53 due to increased UPS activity. In support of this, MDM2 expression was upregulated immediately after p53 reached its maximum level (Supplementary Figure 2a), and MG132-mediated UPS inhibition abrogated the DHA-induced p53 loss at 24 h (Supplementary Figure 2b). These results indicate that DHA-induced p53 is transcriptionally active, confirming its reactivation; on the other hand, they are in line with the notion that DHA also accelerates UPS-dependent p53 degradation. This increased degradation of p53 is not completely unexpected, because we showed that DHA enhances UPS function. Besides p53, DHA also promotes the UPS-dependent degradation of other endogenous UPS substrates, such as *β*-catenin,^35^ the estrogen receptor,^36^ and enhancer of the zeste homolog,^37^ in various cancer cell lines. Although it remains unclear whether the degradation of these proteins results from cellular UPS activation, these observations support the idea that the effect of DHA on UPS modulation may not be restricted to HPV-infected tumor cells.

Prompted by the observation that DHA-induced intracellular ROS primarily localized within mitochondria, we initially examined the contribution of mitochondria-produced superoxide to the total cellular ROS accumulation in DHA-treated HeLa cells. The results showed that DHA increased the mitochondrial ROS level, which was reversed by the antioxidant NAC pretreatment, suggesting that DHA induces cellular ROS by promoting mitochondrial ROS generation. However, in addition to mitochondrial ROS induction, other mechanisms responsible for DHA-induced total ROS accumulation also appear to exist. In support of this idea we found that, whereas NAC pretreatment almost completely blocked the increased level of MitoSOX fluorescence induced by DHA (Figure 5a, NAC+DHA), its inhibitory effect on the intensity of total cellular ROS indicator, DCF, was only partial (Figure 4b, NAC+DHA) when examined under the same conditions (2 h after 50 *μ*M DHA exposure). Previous studies have shown that DHA induces ROS accumulation in a number of malignant cell lines by elevating enzymatical and non-enzymatical lipid peroxidation.^22^ It is therefore likely that DHA may elevate total cellular ROS through some as yet unidentified signaling cascades, which stimulate lipid peroxidation. How DHA exactly affects the mitochondrion to generate high levels of ROS in the organelle is currently not clear; however, our finding that OCR was attenuated immediately upon addition of DHA into the cultures suggests a possible role for DHA, via disruption of mitochondrial electron transport chain, in regulating mitochondrial ROS generation. Indeed, the mitochondrial respiratory chain has long been recognized as a target for unsaturated fatty acid-mediated production of ROS *in vitro* and *in vivo*.^38^

An intriguing finding of this work is that ROS, including mitochondrial-produced and exogenous ROS, repressed E6/E7. We additionally discovered that DHA-induced E6/E7 degradation was attributed to the UPS activation mediated by ROS. Interestingly, despite significant enhancement of proteasome activity in DHA- and H_2_O_2_-treated HeLa cells, such a marked increase was not seen when purified proteasome and cell extracts prepared from HeLa cells were directly exposed to DHA or H_2_O_2_ (Supplementary Figure 5). This suggests that the increased proteasome function observed in cell cultures largely results from an indirect regulatory effect of ROS on the proteasome, probably through induction of Ub-conjugates. Prior work shows that ROS can activate the enzymes involved in ubiquitination,^18, 39^ leading to Ub-conjugates accumulation, which enhances the degradative capacity of the proteasome by stimulating its gate opening.^40^ In these cases, Ub-conjugates formation appears to be an initial step of the ROS-mediated cellular UPS activation, and necessary for the activation of proteasome. In fact, although ROS-mediated UPS activation induced by DHA and H_2_O_2_ was accompanied by the accumulation of Ub-conjugates (Figures 3b and 6b), the attenuated UPS function caused by ROS inhibition was concomitant with decreased levels of Ub-conjugates (Figures 4c and d). These observations indicate a link between Ub-conjugates formation and the UPS function in response to ROS, and it is thus possible that Ub-conjugates may have a role in the ROS-mediated proteasome activation. Nonetheless, although the induction of Ub-conjugates represents one potential mechanism by which ROS indirectly improve proteasome function, results from some recent studies imply that alternative mechanisms may exist. For example, increased proteasome activity caused by interferons is attributed to the ROS-stimulated *de novo* synthesis of inducible proteasome peptidase subunits,^41^ which replace their corresponding conventional peptidase subunits and thereby result in the formation of more functional immunoproteasomes. Further, in a report examining the regulatory effect of Parkin on mitophagy, it was found that mitochondrial dysfunction can act as a signal for proteasome activation and triggers the UPS-dependent clearance of damaged mitochondria.^27^ As increased cellular ROS (including those caused by exogenous application of H_2_O_2_) are known to induce mitochondrial malfunction,^26^ we cannot rule out the possibility that ROS may indirectly enhance proteasome function by triggering mitochondrial failure. Indeed, it had been demonstrated that proteasome activity is correlated with mitochondrial malfunction and oxidative stress in a mouse model of neurometabolic disease, arguing for a role of mitochondria dysfunction in ROS-mediated proteasome activation.^42^ It deserves mention, however, that despite the enhancing effect of ROS on UPS activity observed in this and other studies, impaired UPS function has also been correlated with ROS.^43, 44^ Although not fully clear, these inconsistent results may be partly attributed to the presence of heterogeneous proteasome populations and the differences in experimental conditions, such as the type, dose, and duration of ROS insults.^45^

**Figure 7:**
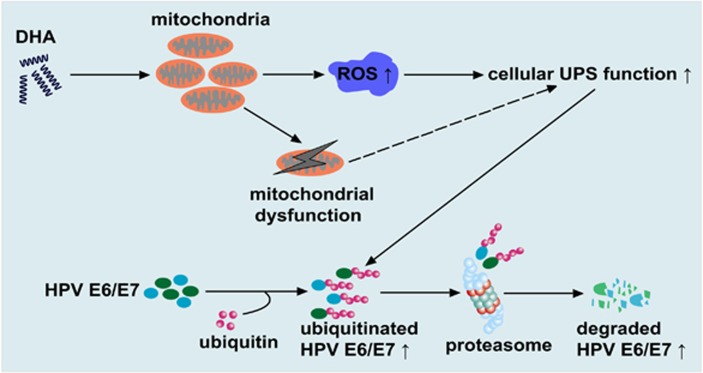
Proposed model showing how DHA reduces the expression of the E6/E7 viral oncoproteins in oncogenic HPV-infected cancer cells. DHA stimulates cellular ROS accumulation primarily via inducing mitochondrial ROS overproduction, which leads to mitochondria failure and the activation of cellular UPS. As a result, the UPS-dependent degradation of E6/E7 viral proteins is accelerated. Note that mitochondrial dysfunction might contribute to the UPS activation induced by DHA (dashed line)

Collectively, the results of the present study identify a unique strategy for inducing the degradation of E6/E7 by activating ROS-mediated UPS function with DHA (Figure 7). Our finding that DHA simultaneously induced UPS activation and E6/E7 degradation highlights a novel biological function of DHA, and reveals one important mechanism by which DHA provokes the death of HPV-associated cancer cells.

## Materials and Methods

### Plasmids, transfection and treatment of stably transfected cells

The expressing plasmids pSG5-HPV-18 E6/E7 (provided by Professor Shih-Ming Huang, Department of Dermatology, Tri-service General Hospital, Taipei, Taiwan) and FLAG-tagged ubiquitin pCS4-FLAG-Ub (provided by Professor Dae-Won Kim, Department of Biochemistry, Yonsei University, Seoul, Korea) were used as reported previously.^46, 47^ The mammalian expression vectors encoding GFP-CL1 and GFP-ODC were kindly provided by Dr. Jong-Bok Yoon (Yonsei University). The Ub^G76V^-GFP construct was a gift of NP Dantuma (Addgene plasmid #11941, Cambridge, MA, USA). Constructs (4−6 *μ*g/10-cm dish) were transfected into cells using Lipofectamine LTX reagent (Invitrogen, Carlsbad, CA, USA; #15338-100) as recommended by the manufacturer. Twelve hours after transfection, the cells were subjected to serum deprivation for 24 h and then subjected to different treatments. HeLa cells stably transfected with Ub^G76V^-GFP plasmids (Ub^G76V^-GFP HeLa) were selected using 0.8 mg/ml G418 as described previously.^15^ For use, Ub^G76V^-GFP HeLa cells were pretreated with the proteasome inhibitor, MG132, to induce the accumulation of the Ub^G76V^-GFP reporter, and then the test compounds were added into the media. To achieve the optimal inhibitory effect of MG132 on the UPS, while avoiding any cytotoxic effects, the cells were pretreated with 2.5 *μ*M MG132 for 4 h followed by a 6-h incubation with the test compounds.

### Immunoprecipitation and western blotting

For immunoprecipitations, HeLa cells transiently transfected with FLAG-tagged ubiquitin vectors were lysed in lysis buffer (25 mM Tris-HCl pH 7.4, 150 mM NaCl, 1% Nonidet P-40, and 5 mM EDTA). Supernatants were added with a HPV-18 E6 antibody or HPV-18 E7 antibody and protein G beads, and incubated at 4 °C for 6 h with shaking. Beads were washed and then boiled in sample buffer for 5 min, and proteins were separated by 15% SDS-polyacrylamide gel electrophoresis for ubiquitination analysis and detection of E6/E7 proteins. Western blotting of whole-cell lysates was performed as described in reference.^23^

### Measurement of oxygen consumption in real time

The OCR was measured using an XF24 Extracellular Flux Analyzer (Seahorse Bioscience, North Billerica, MA, USA). HeLa cells were plated at 2 × 10^4^/well in complete medium and allowed to attach overnight. After washing with serum-free medium, the cells were switched to 590 *μ*l of unbuffered serum-free DMEM containing 25 mM glucose in the absence or presence of 5 mM NAC. Cells were incubated in a CO_2_-free atmosphere at 37 °C for 1 h to allow temperature and pH equilibration before loading onto the XF24 equipment. After three successive 3-min measurements at 5-min intervals, 66 *μ*l of 500 *μ*M DHA (final concentration=50 *μ*M) or solvent vehicle (control) was injected into the corresponding wells. The OCR was monitored by repeating the above measurement cycles 20 times.

### Statistical analysis

The statistical significance of difference between control and treated groups was analyzed using one-way ANOVA. The difference was considered significant when *P*-value was <0.05.

## Abbreviations

CCCP: carbonyl cyanide m-chlorophenylhydrazone
CHX: cycloheximide
CL1: a 16-amino-acid sequence (ACKNWFSSLSHFVIHL) degradation signal peptide
DHA: docosahexaenoic acid
DCF: dichlorodihydrofluorescein
H_2_O_2_: hydrogen peroxide
HPV: human papillomavirus
MDM2: mouse double minute 2 homolog
NAC: *N*-acetyl-cysteine
OCR: oxygen consumption rate
ODC: ornithine decarboxylase
PARP: poly(ADP-ribose) polymerase
Rb: retinoblastoma protein
ROS: reactive oxygen species
Ub-conjugates: ubiquitinated conjugates
UPS: ubiquitin–proteasome system
