# Supplementary figures and images for: Docosahexaenoic acid induces the degradation of HPV E6/E7 oncoproteins by activating the ubiquitin–proteasome system
Source: Cell Death Dis. 2014 Nov 13;5(11):e1524–. doi: 10.1038/cddis.2014.477 (PMC4260735; doi:10.1038/cddis.2014.477)

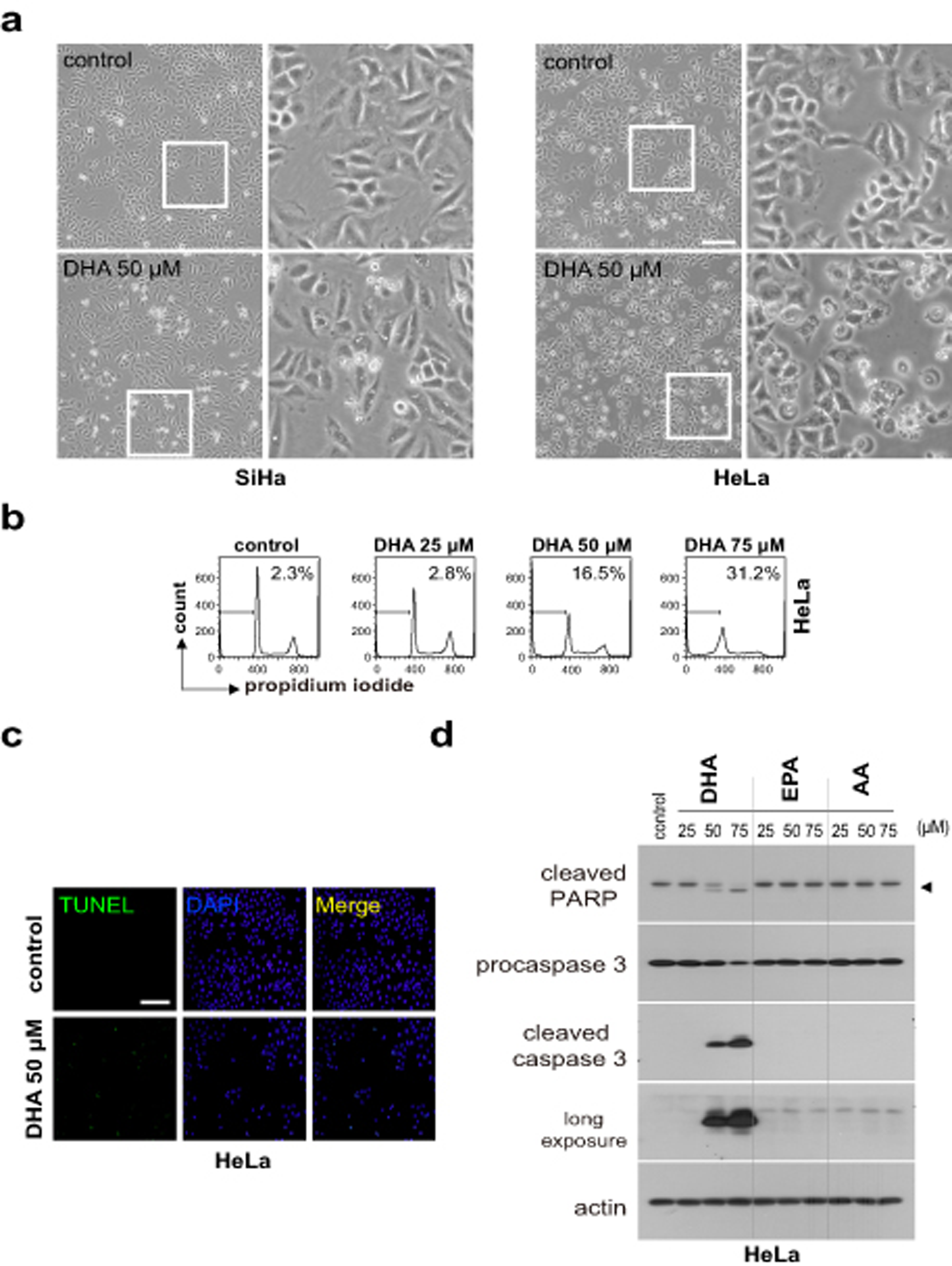

Supplement: Supplementary Figure 1 [file cddis2014477x1.tif]

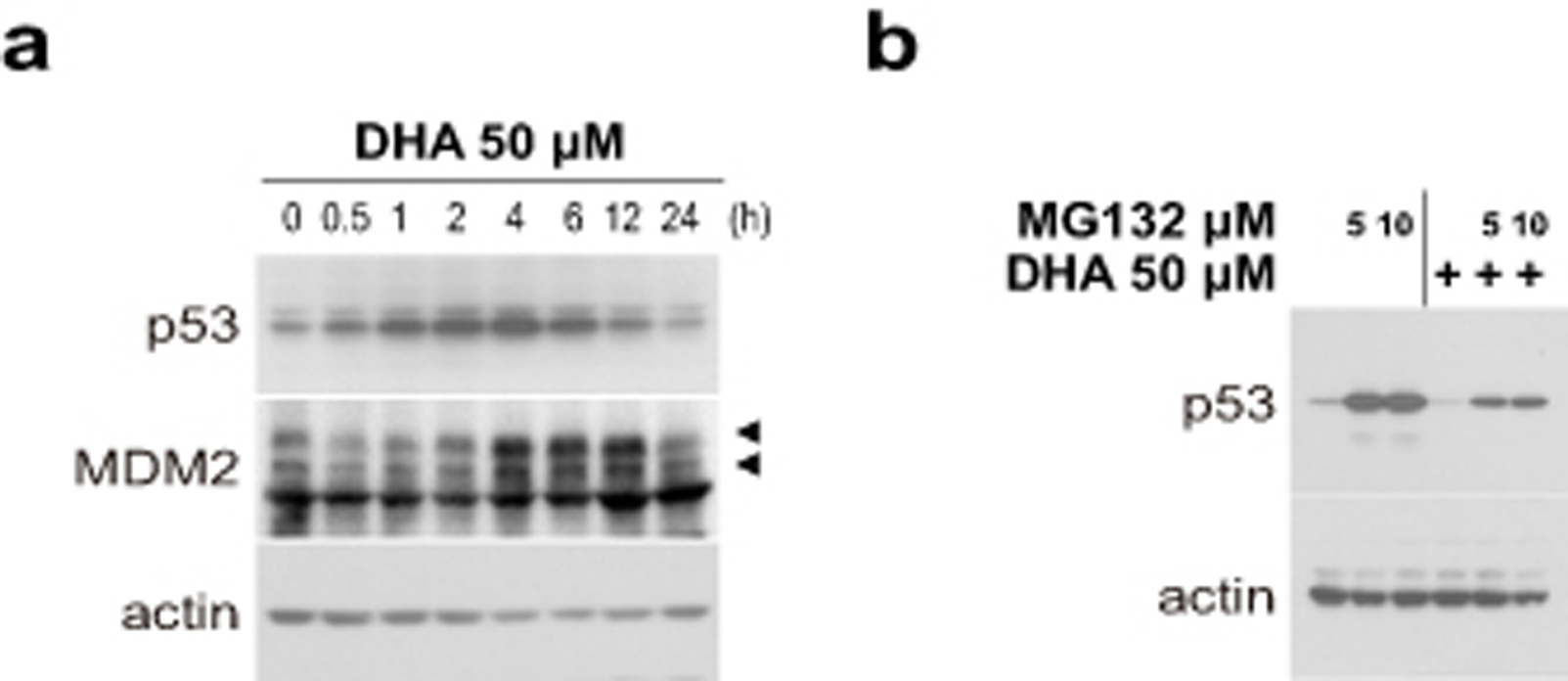

Supplement: Supplementary Figure 2 [file cddis2014477x2.tif]

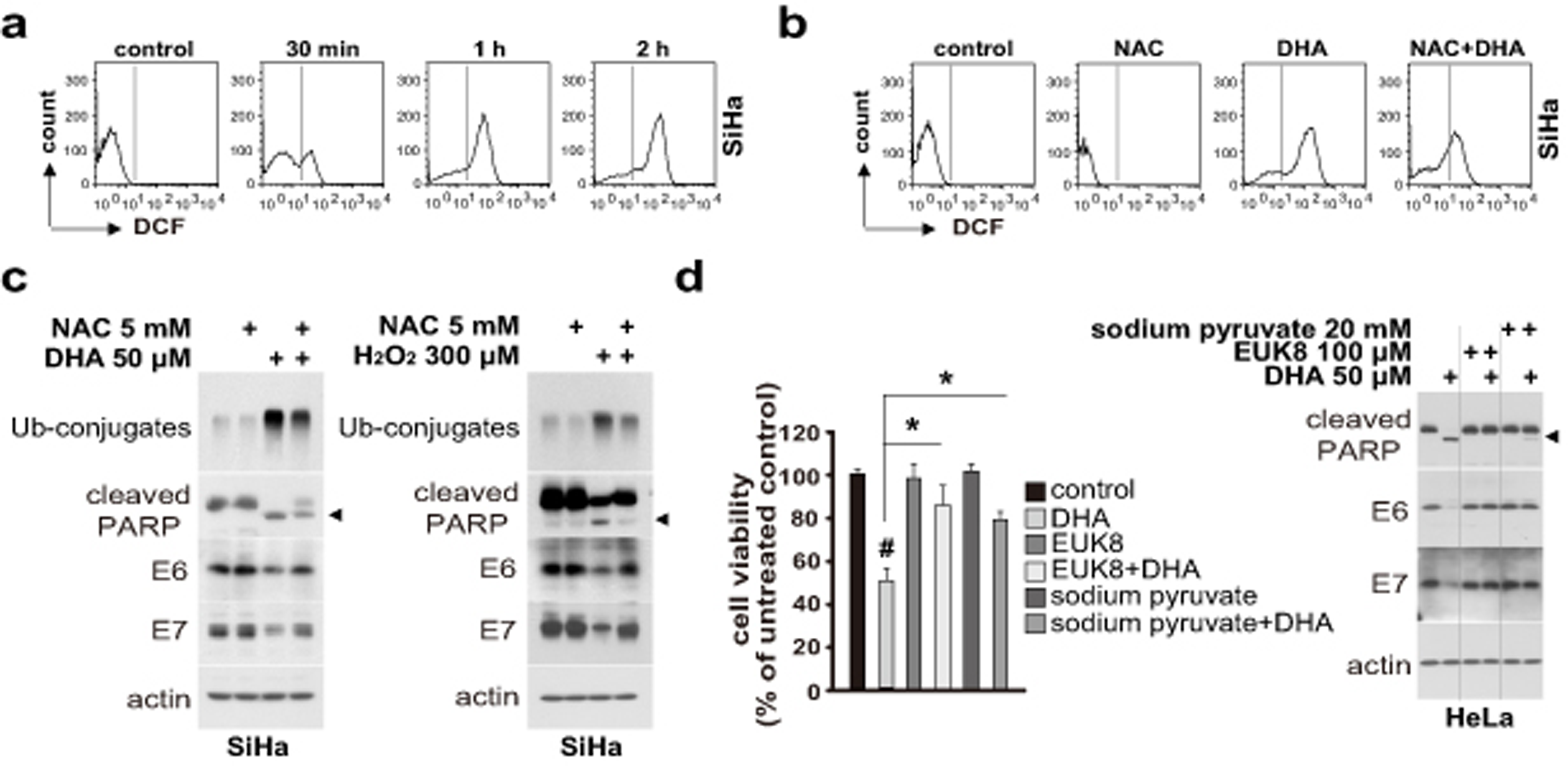

Supplement: Supplementary Figure 3 [file cddis2014477x3.tif]

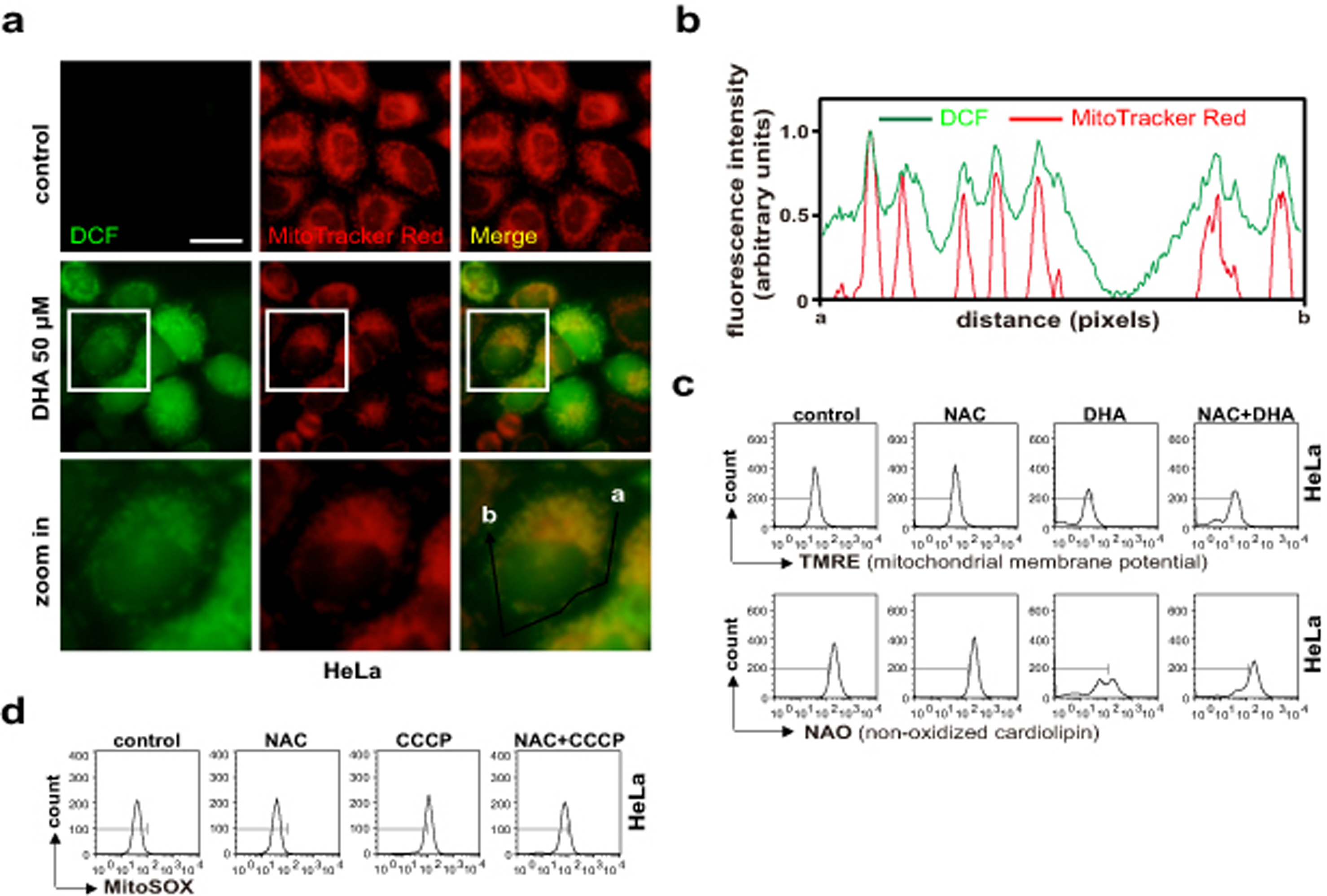

Supplement: Supplementary Figure 4 [file cddis2014477x4.tif]

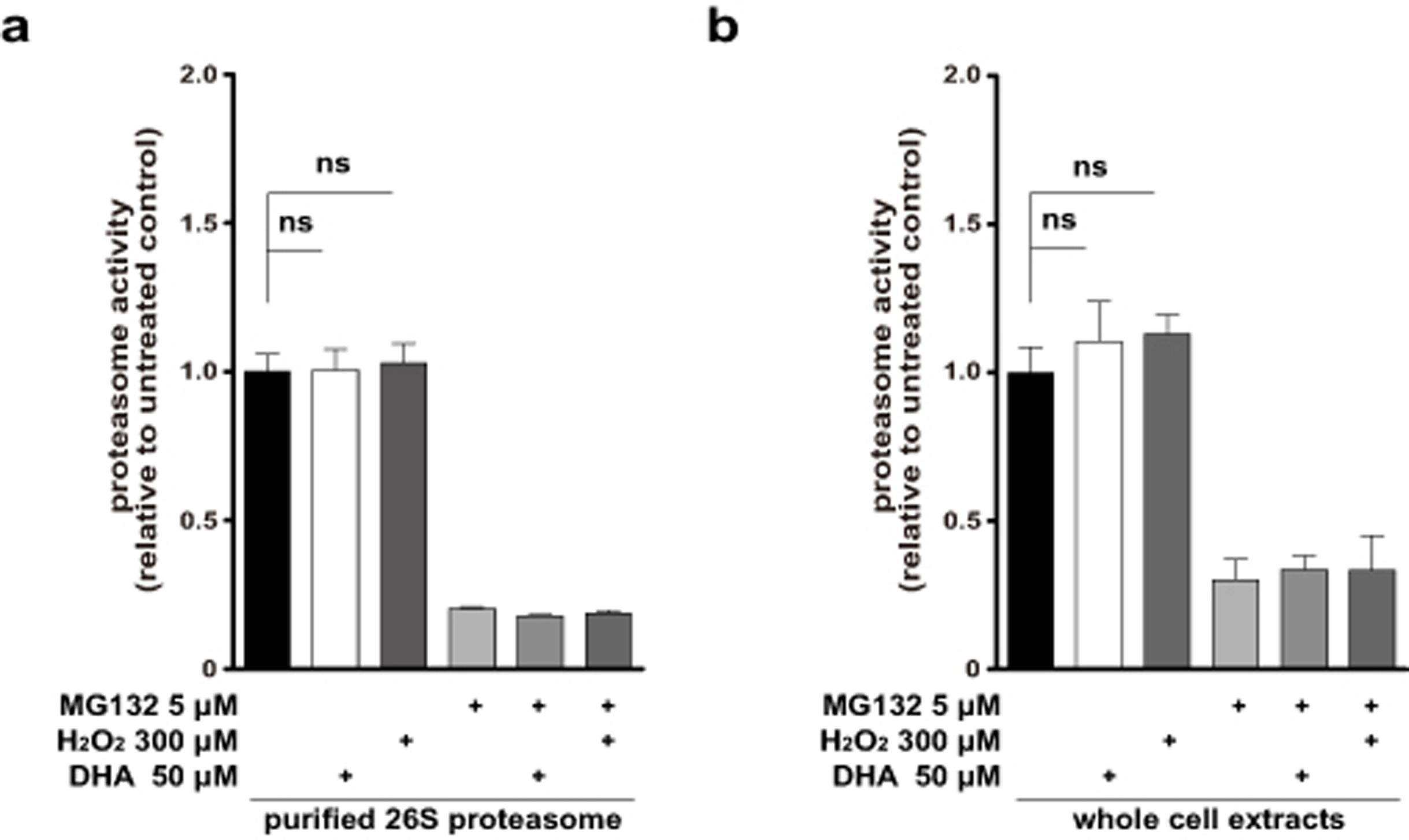

Supplement: Supplementary Figure 5 [file cddis2014477x5.tif]
