# Supplementary material for: Docosahexaenoic acid induces the degradation of HPV E6/E7 oncoproteins by activating the ubiquitin–proteasome system
Source: Cell Death Dis. 2014 Nov 13;5(11):e1524–. doi: 10.1038/cddis.2014.477 (PMC4260735; doi:10.1038/cddis.2014.477)
Supplement: Supplementary Information [file cddis2014477x6.doc]

**Supplementary information**

**Docosahexaenoic acid induces the degradation of HPV E6/E7 oncoproteins by activating ubiquitin-proteasome system**

Kaipeng Jing^1,2,4^, Soyeon Shin^1,2^, Soyeon Jeong^1,2^, Soyeon Kim^1,2^, Kyoung-Sub Song^1^, Ji-Hoon Park^1^, Kang-Sik Seo^1^, Seung-Kiel Park^1^, Gi-Ryang Kweon^1^, Tong Wu^5^, Jong-Il Park^1^ and Kyu Lim^*,1,2,3^

^1^Department of Biochemistry, School of Medicine, ^2^Infection Signaling Network Research Center, and ^3^Cancer Research Institute, Chungnam National University, Daejeon 301-747, Korea; ^4^Stem Cell Research and Cellular Therapy Center, Affiliated Hospital of Guangdong Medical College, Zhanjiang 524001, China; and ^5^Department of Pathology and Laboratory Medicine, Tulane University School of Medicine, New Orleans, LA 70112, USA

**Supplementary Materials and Methods**

**Reagents.** Anti-FLAG anitibodies produced in mouse and rabbit were obtained from Sigma (#F3165 and F7425). Mouse monoclonal antibodies against HPV-16 E6 and HPV-18 E6 were generously provided by the Arbor Vita Corporation (AVC-1006 and AVC-3). Anti-HPV-16 E7 (8C9) antibodies were from Invitrogen. Anti-green fluorescent protein (GFP, #2555) and anti-caspase 3 (#9662) antibodies were from Cell Signaling Technology. Antibodies against poly(ADP-ribose) polymerase (PARP, F-2), HPV-18 E7 (N-19), ubiquitin (FL-76), actin (I-19-R), mouse double minute 2 homolog (MDM2, SMP14), full-length p53 (DO-1) and full-length retinoblastoma tumor suppressor (Rb, C-15) were purchased from Santa Cruz Biotechnology. Goat anti-rabbit, goat anti-mouse and rabbit anti-goat secondary antibodies were obtained from Calbiochem (#401315, 401215 and 401515). Protein G-agarose beads (#11719416001) and 4',6-diamidino-2-phenylindole (DAPI, #10236276001) were provided by Roche. Docosahexaenoic acid (DHA, #90310), eicosapentaenoic acid (EPA, #90110) and arachidonic acid (AA, #90010) were from Cayman Chemical, and prepared as 100 µM stock solutions in absolute ethanol before use (final ethanol concentration was < 0.05% vol/vol). Proteasome inhibitors (lactacystin, MG132 and MG262) and G418 were purchased from Merck Millipore (#426100, 474790 and 539163) and Cellgro (#61-234), respectively. Purified 26S proteasome from HeLa cells and fluorogenic proteasome substrates, Suc-LLVY-Aminomethylcoumarin (AMC) were kindly provided by Prof. Min Jae Lee (Kyung Hee University, Yongin, Korea). Chloromethyl dichlorodihydrofluorescein diacetate (CM-H2DCFDA, #C6827), 10-N-nonyl acridine orange (NAO, #A1372), tetramethylrhodamine ethyl ester (TMRE, #T669), MitoTracker Red (#M22426) and MitoSOX Red (#M36008) were from Molecular Probes. All other chemicals were obtained from Sigma unless otherwise stated.

**Cell culture.** Human cancer cell lines SiHa (HPV-16-positive), HeLa (HPV-18-positive) and A549 cells (HPV-negative) were purchased from American Type Culture Collection (HTB-35, CCL-2 and CCL-185). All the cells and their transiently or stably transfected derivatives were maintained in Dulbecco's modified Eagle's medium (Invitrogen, #31600) supplemented with 10% fetal bovine serum (Invitrogen, #10082147) and 1% penicillin-streptomycin (Invitrogen, #15140-122). Cells grown to 70% confluency were switched to serum-free medium and the culture was allowed to expand for 24 h before giving any treatment.

**Cell viability and apoptosis assays.** MTT (thiazolyl blue tetrazolium bromide, Sigma, #M2128) assays were used to examine the viability of HeLa cells, SiHa cells, and HPV-negative A549 lung cancer cells transfected with the pSG5-HPV-18 E6/E7 plasmids. HeLa or SiHa cells (7 × 10^3^) were seeded into the wells of a 96-well plate. The cells were cultured overnight in serum-supplemented medium, and then switched into serum-free medium for 24 h before treatment with DHA. A549 cells were seeded onto 96-well plates (1 × 10^4^ cells/well). Twelve hours after transfection, they were subjected to serum deprivation for 24 h before treatment with DHA. At the end of the treatment, MTT was added to each well (final concentration 200 µg/mL) and the cultures were incubated at 37°C for 1 h. The absorbance was measured at 570 nm in a microplate spectrophotometer (Thermo Fisher Scientific), and cell viability was expressed as a percentage relative to untreated control cells. Apoptosis was monitored by the terminal deoxyribonucleotidyl transferase-mediated dUTP nick end labeling (TUNEL) method for detecting DNA strand breaks, and the Sub-G1 method for identifying cells with fractional DNA content (Sub-G1 DNA content). For TUNEL assay, HeLa cells were stained using the DeadEnd™ Fluorometric TUNEL System (Promega, #G3250) followed by nuclear counterstaining with DAPI. For Sub-G1 analysis, both attached and floating cells were collected, stained with propidium iodide (Sigma, #P4170), and assessed by flow cytometry (FACS-Calibur, BD Biosciences).

**Fluorescence analysis of reactive oxygen species (ROS) production and mitochondrial function.** Cells seeded onto 6-well plates were first incubated with CM-H2DCFDA (2.5 μM), MitoSOX Red (2.5 μM), TMRE (25 nM) or NAO (5 μM) in Hanks' balanced salt solution (HBSS, Sigma, #H8264) for 30 min. The stained cells were washed twice with HBSS, and incubated with test compounds in serum-free medium. Cells were then collected, suspended in 500 μL HBSS, and the fluorescence intensity was assessed by flow cytometry. For imaging of vital mitochondria, cells were grown on coverslips, treated with the different compounds, and then stained with MitoTracker Red (50 nM) for 20 min at 37°C. The cells were then fixed and observed under an Olympus FV500 confocal microscope. For colocalization between DCF and MitoTracker Red particles, line profiles from the two fluorescent channels on the merged images were plotted using Image J software. Briefly, MitoTracker Red fluorescent particles were randomly picked from the red channel, and double-channel line profiles were drawn for the same particle in the corresponding merged image. The line profile implies the distribution of the two fluorophores on the drawn line analyzed. The y-axis represents the fluorescence intensity values (in arbitrary units), and x-axis is the distance (in pixels) along the line.

**Immunofluorescence staining.** HeLa cells grown on coverslips were fixed in 4% paraformaldehyde (Fisher, #NC9245948) at room temperature for 10 minutes. After blocking with 1% bovine serum albumin (BSA) for 1 h, cells were incubated with mouse anti-p53 (1:300) or rabbit anti-Rb (1:500) primary antibodies at 4°C overnight. Donkey anti-mouse and anti-rabbit secondary antibodies were Alexa 488 green-conjugated (Molecular Probes, #A-21202 and A-21206). After nuclear counterstaining with DAPI, the cells were observed under an Olympus iX70 inverted fluorescence microscope using DP Controller software (Olympus) for image acquisition.

***In vitro* proteasome activity assays.** Peptidase activities were determined by measuring the predominant chymotrypsin-like activity of 26S proteasome, using fluorogenic proteasome substrates Suc-LLVY-AMC. To assess the effects of test compounds on 26S proteasome function in a context close to the cellular milieu, HeLa cells treated with or without test compounds were pelleted at 1,000 rpm, washed twice with phosphate buffered saline, resuspended in lysis buffer (100 mM NaCl, 50 mM NaH_2_PO_4_ pH 7.5, 10% glycerol, 5 mM MgCl_2_, 0.5% Nonidet P-40, 5 mM ATP, 1 mM dithiothreitol (DTT) and protease inhibitor cocktail) and lysed by passing ten times through a 27-gauge needle attached to a 1-mL syringe on ice. Lysates were centrifuged at 1,2000 rpm for 15 min at 4 °C, and after collecting the supernatant, protein concentration of the cell homogenates was determined using the Bradford assay (Bio-Rad, #500-0006). Ten microliters of cell lysates containing equal amounts of proteins (10 μg) were aliquoted to a 96-well black plate, and incubated with 85 μL of proteasome assay buffer (50 mM Tris-HCl pH 7.5, 1 mM EDTA, 1 mg/mL BSA, 1 mM ATP and 1 mM DTT) for 10 min on ice before 5 μL of 250 μM Suc-LLVY-AMC substrates were added. Fluorescence was monitored on a microplate fluorometer (Twinkle LB970, Berthold Technologies) using 355/460 nm filter sets at 37 °C. Background signals detected from the wells which only contain the substrates and assay buffer, were subtracted from each analysis for data presentation. The mean fluorescence value of untreated control group was set to 1, and used to normalize the other treatment groups. To examine the direct effects of test compounds on 26S proteasome function, a total volume of 95 μL reaction mixture in the proteasome assay buffer containing 26S proteasome purified from HeLa cells (125 ng) and test compounds (or vehicles) was incubated in 96-well black plates on ice for 10 min before 5 μL of 250 μM Suc-LLVY-AMC substrates were added. Detection and quantification of the liberated AMC was performed as outlined above. All reactions were run in triplicates.

**Supplemental Figure Legends**

**Supplementary Figure 1** DHA, but not eicosapentaenoic acid (EPA) and arachidonic acid (AA), induces apoptosis in oncogenic HPV-infected cancer cells. (**a**) Bright field microscopic images of SiHa (left) and HeLa (right) cells treated with or without 50 µM DHA for 12 h. Right panel is taken from the left panel insets, respectively. Note the presence of multiple cytoplasmic vecuoles and membrane blebs in the cell cultures treated with DHA (scale bar, 100 μm). (**b**) HeLa cells were left untreated (control) or treated with 25, 50 and 75 µM DHA for 6 h, and the portion of cells exhibiting Sub-G1 phase DNA (nuclear fragments) was examined using propidium iodide by flow cytometry. (**c**) HeLa cells were treated with or without 50 µM DHA for 12 h, and subjected to the TUNEL assay for detecting DNA strand breaks followed by DAPI nuclear counterstaining. Scale bar, 200 μm. (**d**) HeLa cells were incubated with indicated concentrations of DHA, EPA or AA for 6 h, and the expression levels of PARP and caspase 3 were assessed by Western blot.

**Supplementary Figure 2** Long-term exposure to DHA represses p53 expression by increasing its proteasomal degradation. (**a**) HeLa cells were incubated with 50 µM DHA for indicated times, and the protein levels of p53 and its transcriptional target, MDM2, were analyzed by Western blotting. (**b**) HeLa cells were left untreated or incubated with 50 µM DHA for 24 h with or without 1 h of indicated doses of MG132 pretreatment, and the whole cell lysates were blotted for p53.

**Supplementary Figure 3** DHA-induced reduction in E6/E7 expression involves ROS. (**a**) SiHa cells were treated with 50 µM DHA for indicated times, and intracellular ROS levels were detected by flow cytometry using CM-H2DCFDA probes. (**b**) CM-H2DCFDA-loaded SiHa cells were treated with 50 μM DHA for 2 h with and without 1 h of pretreatment of 5 mM NAC, and the ROS levels were detected by flow cytometry. (**c**) SiHa cells were incubated with 50 μM DHA (left) or 300 µM H_2_O_2_ (right) for 6 h with and without 1 h of 5 mM NAC pretreatment, and whole cell lysates were blotted with the indicated antibodies. (**d**) HeLa cells were left untreated or treated with 50 μM DHA for 6 h with 1 h of pretreatment of EUK8 or sodium pyruvate. Cell viability was measured by MTT assays (left), or cells were subjected to immunoblotting (right).

**Supplementary Figure 4** Effect of DHA and CCCP on mitochondrial ROS production. (**a**) Fluorescence micrographs of CM-H2DCFDA-loaded HeLa cells stained with MitoTracker Red. The cells were left untreated or treated with 50 μM DHA for 20 min, and then stained with 50 nM MitoTracker Red (scale bar, 10 μm). The black square box area is magnified in the panel labeled as “zoom in” and the arrow path indicates the fluorescent signal from DCF and MitoTracker shown in (**b**). (**b**) Line profiles on the merged image were plotted for the relative fluorescent intensity values of green and red channels against the pixel position along the line scan. (**c**) HeLa cells were left untreated, or incubated with 50 μM DHA for 2 h with and without 5 mM NAC pretreatment for 1 h. The levels of mitochondrial membrane potential (top) and non-oxidized cardiolipin form mitochondrial membranes (bottom) were monitored by flow cytometry using tetramethylrhodamine ethyl ester (TMRE) or 10-N-nonyl acridine orange (NAO) probes, respectively. (**d**) HeLa cells were left untreated, or incubated with 1 μM CCCP for 2 h with and without 1 h of pretreatment of 5 mM NAC, and mitochondrial ROS levels were assessed by flow cytometry using MitoSOX Red. Data are represented as mean ± SD values, and error bars indicate SD. *p < 0.05; #p < 0.001 (n=3).

**Supplementary Figure 5** Degradation activity of the proteasome is not directly affected by DHA and H_2_O_2_. (**a**, **b**) Suc-LLVY-AMC substrates were incubated with 26S proteasome (**a**) or whole cell extracts (**b**) prepared from HeLa cells in the presence vehicle solvent (untreated control), 50 μM DHA, 300 µM H_2_O_2_ or 5 μM of proteasome inhibitor MG132 (positive control) for 1 h, and the fluorescence signal of released AMC was measured. The effect of treatment with MG132 is also shown, and the untreated control was set to 1. The results are expressed as the mean ± SD of three experiments (ns, nonsignificant difference).
